# Clinical Differences Between Single and Multiple Suicide Attempters, Suicide Ideators, and Non-suicidal Inpatients

**DOI:** 10.3389/fpsyt.2020.605140

**Published:** 2020-12-15

**Authors:** Isabella Berardelli, Alberto Forte, Marco Innamorati, Benedetta Imbastaro, Benedetta Montalbani, Salvatore Sarubbi, Gabriele Pasquale De Luca, Martina Mastrangelo, Gaia Anibaldi, Elena Rogante, David Lester, Denise Erbuto, Gianluca Serafini, Mario Amore, Maurizio Pompili

**Affiliations:** ^1^Department of Neurosciences, Mental Health, and Sensory Organs, Faculty of Medicine and Psychology, Suicide Prevention Centre, Sant'Andrea Hospital, Sapienza University of Rome, Rome, Italy; ^2^Department of Human Sciences, European University of Rome, Rome, Italy; ^3^Psychiatry Residency Training Program, Faculty of Medicine and Psychology, Sant'Andrea Hospital, Sapienza University of Rome, Rome, Italy; ^4^Department of Psychology, Sapienza University of Rome, Rome, Italy; ^5^Psychology Program, Stockton University, Galloway, NJ, United States; ^6^Department of Neuroscience, Rehabilitation, Ophthalmology, Genetics, and Maternal and Child Health, Psychiatry Section, University of Genoa, IRCCS San Martino, Genoa, Italy

**Keywords:** suicide, single suicide attempters, multiple suicide attempters, suicide ideators, lethality

## Abstract

Single suicide attempters (SSAs) and multiple suicide attempters (MSAs) represent distinct subgroups of individuals with specific risk factors and clinical characteristics. This retrospective study on a sample of 397 adult psychiatric inpatients analyzed the main sociodemographic and clinical differences between SSAs and MSAs and the possible differences between SSAs, MSAs, and psychiatric patients with and without suicidal ideation (SI). Clinical variables collected included psychiatric diagnoses (Mini International Neuropsychiatric Interview), presence of substance use, current suicide risk status (Columbia Suicide Severity Rating Scale), Clinical Global Impression at admission, Global Assessment of Functioning improvement between admission and discharge, age at onset of psychiatric illness, duration of untreated illness in years, number of hospitalizations in psychiatric settings, and lethality of the most severe suicide attempt. A multinomial logistic regression model with groups showed that MSAs had a higher lethality of their last suicide attempt as compared to SSAs. In addition, MSAs had distinct sociodemographic characteristics compared to both SSAs and patients with SI. Although the study was limited by the relatively small sample size and retrospective nature, the present results suggest that identifying MSAs could be useful in predicting suicide risk and designing *ad hoc* prevention strategies.

## Introduction

Suicide is a major public health issue, and nonfatal suicidal behaviors are lethal risk factors for suicide (1). World statistics indicate that for every suicide death, there are up to 25 suicide attempts (2). Bostwick et al. (1) found that ~60% of individuals who completed suicide died on their index attempt (i.e., their first-lifetime attempt that required medical attention). Among the 40% of suicide attempters who died as a result of their second or later attempt, more than 80% died within a year of the initial attempt. The study by Isometza and Lonnqvist (3) found that the majority of those who died by suicide (56%) died on their first attempt. In people who attempted suicide multiple times, Isometza and Lonnqvist also reported sex differences and a change in suicide methods, which likely increased the lethality of subsequent suicidal behavior. A high lethality of nonfatal attempts is an important predictor of later suicide, and multiple self-harm acts alone increase subsequent suicide risk (4). In successful final suicide attempts that involved hanging and gas poisoning (mainly charcoal burning), there was a tendency to adopt the same method as the last survived event, though this phenomenon is less marked for suicide attempters who survived jumps, overdoses, and cutting (5). Despite the above findings, the impact of suicide attempt frequency and level of lethality on suicide mortality remains unclear.

In comparison to single suicide attempters (SSAs), multiple suicide attempters (MSAs) likely represent a distinct subgroup of individuals with specific risk factors and clinical characteristics (6–11). For example, MSAs usually manifest borderline personality traits (8, 10, 12). Forman et al. (10) reported more severe depressive symptoms, suicidal ideation (SI), and hopelessness in MSAs than in SSAs, even after controlling for the diagnosis of borderline personality disorder. Furthermore, factors such as a family history of suicidal behavior, poor coping skills, and more severe psychopathology were more strongly associated with MSAs than SSAs (7).

As compared to SSAs, MSAs also seem to exhibit a greater number of suicide risk factors (e.g., a history of childhood emotional abuse and family suicide), increased psychopathology (e.g., depression and substance abuse), higher levels of suicidality (e.g., SI), and lower interpersonal functioning. As compared to SSAs, MSAs had significantly higher motor impulsivity, indicating spur-of-the-moment action (13, 14). MSAs also had a longer duration of bipolar illness, more frequently lived alone, had more than one psychiatric comorbidity, and were more likely to attempt suicide by self-poisoning (although this is also the most common suicide method in SSAs) (15). MSAs and SSAs presented higher levels of depression, hopelessness, aggression, hostility, impulsivity, borderline personality traits, and family history of major depression or alcohol use disorder as compared to psychiatric patients who were non-attempters (16). Despite these findings, some debate exists as to whether MSAs and SSAs have different clinical characteristics. Paashaus et al. (17) compared subjects with SI, MSAs, and SSAs in order to evaluate suicide capability, conceptualized by Joiner (18) as fearlessness about death, subjective pain tolerance, and objective pain persistence, and found no significant differences. Previous research also indicated differences and similarities between SSAs and subjects with SI (19).

In the present study, aimed to identify differences in sociodemographic factors and clinical features between SSAs and MSAs, we hypothesized that SSAs, MSAs and psychiatric patients with and without SI are a distinct population of patients.

## Materials and Methods

### Participants

We enrolled 397 adult inpatients (202 men and 195 women) consecutively admitted to the University Psychiatric Clinic, Sant'Andrea Hospital, Sapienza University of Rome between 2017 and 2019. In the period of the study (2017–2019) the number of patients admitted to the hospital was 800. The mean age of participants was 40.41 years [standard deviation (SD) = 14.06; age range = 17–78 years]. Sociodemographic and clinical characteristics of the sample are summarized in Table 1. Inclusion criteria were: (1) Diagnostic and Statistical Manual of Mental Disorders (DSM)-5 criteria for psychiatric disorders, and (2) informed consent for participation in the study. Exclusion criteria were: (1) severe neurological disorders (epilepsy, cognitive impairment, or genetic syndromes), and (2) the presence of cognitive deficits causing linguistic problems.

**Table 1:** Sociodemographic and clinical characteristics.

**Variables**	***N***	**%**
**Sex**		
Male	202	50.9
Female	195	49.1
Age—M|SD	40.41|14.06	
**Marital status**		
Married	110	27.8
Divorced or widowed	50	12.6
Single	236	59.6
**Housing**		
Living with family or others	303	76.5
Living alone	68	17.2
Other	25	6.3
**Job**		
Employed	167	42.1
Unemployed	201	50.6
Retired or other	29	7.3
**Educational achievement**		
≤ 8 years^a^	141	35.5
=13 years^b^	188	47.4
≥16 years^c^	68	17.1
**DSM-5 diagnosis**		
Major depressive disorder	47	12.1
Bipolar disorder	161	41.6
Schizophrenia or other psychoses	109	28.2
Personality disorder	39	10.1
Other	31	8.0
Comorbidity (yes)	28	7.1
Current admission for suicide attempt	123	31.0
**Suicide risk**		
Single attempters (current)	58	14.6
Multiple attempters	65	16.4
Suicide ideators	57	14.4
No attempts/ideation	217	54.7
CGI—M|SD	4.47	1.97
GAF improvement—M|SD	25.72	19.17
Age at onset—M|SD	26.48	12.64
Number of hospitalizations—M|SD	1.76	2.36
DUI—M|SD	3.45	7.22
Lethality of the most severe suicide attempt—M|SD	1.91	1.07

a *Middle school*,

b *High school*,

c *Master degree*.

All patients participated voluntarily and gave their informed consent to participate in the study. The study analyzed baseline characteristics as part of a broader investigation on mental pain and suicide risk approved by the local institutional review board.

### Measures

Two independent psychiatrists at the University Psychiatric Clinic, Sant'Andrea Hospital, Sapienza University of Rome analyzed each clinical record. Data were reported on a structured checklist created for this study by the authors. In cases of disagreement between the two evaluators, a third party was consulted. The k value for interrater reliability was 0.96.

Clinical variables collected included psychiatric diagnoses, the presence of substance abuse, current suicide risk status, Clinical Global Impression (CGI) (20) at admission, Global Assessment of Functioning (GAF) (21) improvement between admission and discharge, age at onset of psychiatric illness, duration of untreated illness (DUI) in years, number of hospitalizations in psychiatric settings, and lethality of the most severe suicide attempt (22).

Psychiatric diagnosis was based on the Diagnostic and Statistical Manual of Mental Disorders, fifth edition (23) and supported bythe Italian translation of the Mini International Neuropsychiatric Interview (MINI). The MINI is a short, structured interview developed in France and the United States to explore 17 disorders according to the DSM-III-R (24). It has undergone many reliability and validity studies (25) and has been updated to map both DSM-IV, MINI 6.0 (version 10/10/10) and DSM-5, MINI 7.0.2 diagnostic criteria.

SI and suicide attempts were assessed using the Italian version of the Columbia Suicide Severity Rating Scale (C-SSRS) (26) administered by psychiatric residents in the first 2 days after admission to the psychiatric department. The C-SSRS was used to assess SI severity and intensity, types of suicidal behavior, and lethality of suicide attempts at time points and overtime periods. Use of the C-SSRS differentiated four different patient groups: SSAs, MSAs, and two control groups of non-attempters: a group of psychiatric patients with no recorded suicide risk and a group of psychiatric patients who reported SI but no current or lifetime suicide attempts. The C-SSRS begins with two items that assess the respondent's wish to be dead (e.g., “I wish I were dead”) and nonspecific active suicidal thoughts (e.g., "I've thought about killing myself”). If the participant responds affirmatively to either of these two items, they are presented with three additional items that assess active SI with any method but with no plan or intent to act; active SI with some intent to act but no plan; and active SI with a specific plan and intent. The assessment of active SI is therefore conditioned on the individual's endorsement of the wish to be dead and/or nonspecific active suicidal thoughts, based on the assumption that more severe forms of SI subsume less severe forms of SI. Given the difficulties in categorizing suicide attempts, we also referred to the revised nomenclature in suicidology (27, 28). We, therefore, referred to suicidal acts that were assessed in patients included in this sample as type-II suicide attempts, which may be described as self-destructive acts with some degree of intent to end one's life and some identifiable injuries.

Based on C-SSRS scores, we differentiated the lethality of suicide attempts as follows according to actual lethality/medical damage: 0: no physical damage or very minor physical damage (e.g., surface scratches); 1: minor physical damage (e.g., lethargic speech, first-degree burns, mild bleeding, sprains); 2: moderate physical damage, medical attention needed (e.g., conscious but sleepy, somewhat responsive, second-degree burns, bleeding of a major vessel); 3: moderately severe physical damage, medical hospitalization and intensive care likely required (e.g., comatose with reflexes intact, third-degree burns on <20% of the body, extensive blood loss but can recover, major fractures); 4: severe physical damage, medical hospitalization with intensive care required (e.g., comatose without reflexes, third-degree burns over 20% of the body, extensive blood loss with unstable vital signs, major damage to a vital area); and 5: death.

### Statistical Analysis

All statistical analyses were performed with the statistical package for social sciences (SPSS 19.0). A series of ANOVAs and chi-square (χ^2^) tests were used for bivariate analyses. Significant variables in bivariate analyses were included as independent variables in a multinomial regression analysis model with groups as a criterion. Odds ratios (ORs) and their 95% confidence intervals (CIs) were reported as measures of association. Tamhane's T2 *post-hoc* tests were used for group comparison. All tests were considered statistically significant if *p* < 0.05.

## Results

### Group Characteristics

Fifty-eight patients had attempted suicide in the past few days (SSAs), 65 patients had attempted suicide in the last few days and had also attempted suicide in the past (MSAs), 57 patients reported lifetime SI but not behaviors, and 217 patients did not report either lifetime SI or behaviors (Table 1). Twelve percent of patients had unipolar major depression, 41.6% had bipolar disorder, 28.2% had schizophrenia or other psychoses, 10.1% had a personality disorder, and 8.0% had other specified disorders (mainly anxiety disorders). Twenty-eight patients also reported comorbidities with at least one other disorder (7.1% with mainly personality and anxiety disorders) and 23 reported substance abuse (7.0%) (Table 1).

### Difference Between Groups

The four groups differed according to marital status ($\chi_{6}^{2}$ = 19.77, *p* = 0.003), diagnosis ($\chi_{12}^{2}$ = 31.39, *p* = 0.002), GAF improvement during the last hospitalization (F_3;393_ = 251.25, *p* < 0.001), number of hospitalizations (F_3;393_ = 3.70, *p* = 0.012), and DUI (years) (F_3;393_ = 4.25, *p* = 0.006) (see Table 2). The four groups did not differ in terms of sex, age, job, educational achievement, housing, psychiatric comorbidities, substance abuse, CGI scores, or age at onset of psychiatric disorder (Table 2).

**Table 2:** Differences between subgroups.

	**Single attempters (current) *N* = 58**	**Multiple attempters *N* = 65**	**Suicide ideation, no attempt *N* = 57**	**No attempts/ideation *N* = 217**	**Test**	***p*-value**
Sex					*χ^2^*_3_ = 1.38	0.71
Male	46.6%	47.7%	56.1%	51.6%		
Female	53.4%	52.3%	43.9%	48.4%		
Age—M|SD	42.95	40.51	37.60	40.43	*F*_3;393_ = 1.40	0.24
	14.96	14.60	12.30	14.03		
Marital status					*χ^2^*_6_ = 19.77	0.003
Married	48.3%	26.2%	24.6%	23.6%		
Divorced or widowed	12.1%	20.0%	8.8%	11.6%		
Single	39.7%	53.8%	66.7%	64.8%		
Job					*χ^2^*_6_ = 4.61	0.59
Employed	44.8%	40.0%	47.4%	40.6%		
Unemployed	43.1%	53.8%	49.1%	52.1%		
Retired or other	12.1%	6.2%	3.5%	7.4%		
Educational achievement					*χ^2^*_6_ = 5.27	0.51
≤ 8 years	32.8%	40.0%	24.6%	37.8%		
13 years	46.6%	44.6%	52.6%	47.0%		
≥16 years	20.7%	15.4%	22.8%	15.2%		
Housing					*χ^2^*_6_ = 4.61	0.60
Living with family or others	82.8%	72.3%	83.9%	74.2%		
Living alone	13.8%	20.0%	12.5%	18.4%		
Other	3.4%	7.7%	3.6%	7.4%		
DSM-5 Diagnosis					*χ^2^*_12_ = 31.39	0.002
Major Depressive Disorder	14.5%	17.7%	11.1%	10.2%		
Bipolar Disorder	34.5%	38.7%	37.0%	45.4%		
Schizophrenia and other psychoses	30.9%	24.2%	25.9%	29.2%		
Personality Disorder	20.0%	17.7%	11.1%	5.1%		
Other	0.0%	1.6%	14.8%	10.2%		
Comorbidities	6.9%	12.3%	5.3%	6.0%	*χ^2^*_3_ = 3.39	0.34
CGI—M|SD	4.24	4.34	5.08	4.43	*F*_3;393_ = 2.00	0.11
	0.73	0.96	5.07	0.79		
GAF improvement—M|SD	49.17	48.52	13.00	15.96	*F*_3;393_ = 251.25	<0.001
	12.87	14.05	8.97	10.38		
Age at onset—M|SD	26.95	24.48	24.43	27.54	*F*_3;393_= 1.55	0.20
	14.12	10.79	10.75	13.15		
Number of hospitalizations—M|SD	0.95	1.84	1.47	2.06	*F*_3;393_= 3.70	0.012
	1.55	2.17	1.77	2.69		
Duration of Untreated Illness—M|SD	6.22	3.72	3.80	2.49	*F*_3;393_= 4.25	0.006
	10.27	6.12	6.88	6.39		
Lethality of the most severe suicide attempt—M|SD	1.50	2.28	–	–	*t*_109.24_ = −4.24	<0.001
	1.11	0.89				
Methods					*χ^2^_4_= 6.805*	*0.147*
Cut/Pierce	23.3%	5.1%	–	–		
Drug ingestion	50.0%	51.3%	–	–		
Hanging	10.0%	7.7%	–	–		
Jump	13.3%	25.6%	–	–		
Poison by gas	3.3%	10.3%	–	–		

*χ^2^ = mean Chi square value; F = mean Fisher value; t = mean t test value.*.

SSAs were more frequently married than other groups (48.3 vs. 26.2, 24.6, and 23.6%, respectively, for MSAs, patients with SI, and controls), and less frequently single (39.7 vs. 53.8, 66.7, and 64.8%, respectively, for MSAs, patients with SI, and controls). MSAs were more frequently divorced/widowed than other groups (20.0 vs. 12.1, 8.8, and 11.6%, respectively, for SSAs, patients with SI, and controls). SSAs and MSAs were more likely to have higher GAF improvement during the last hospitalization (49.17 ± 12.87 and 48.52 ± 14.05 vs. 13.00 ± 8.97 and 15.96 ± 10.38, respectively, for patients with SI and controls), and SSAs were more likely to have had fewer hospitalizations (0.95 ± 1.55 vs. 2.06 ± 2.69) and a longer DUI (years) (6.22 ± 10.27 vs. 2.49 ± 6.39) than controls. Suicide attempters also more frequently had a personality disorder diagnosis as compared to controls with no known suicide risk (20.7 and 18.5% for MSAs and SSAs, respectively, vs. 6.5% for controls when considering major diagnoses and comorbidities; $\chi_{3}^{2}$ = 13.54, *p* = 0.004).

Thus, MSAs and SSAs differed according to marital status and lethality of the most severe suicide attempt (evaluated with the C-SSRS), with higher lethality observed in MSAs (1.50 ± 1.11 and 2.28 ± 0.89 for SSAs and MSAs, respectively; *t*_109.24_ = −4.24, *p* < 0.001).

Suicide attempters differed from control groups according to marital status, GAF improvement during the last hospitalization, number of hospitalizations, and DUI (the latter differed only between SSAs and controls).

**Figure 1:**
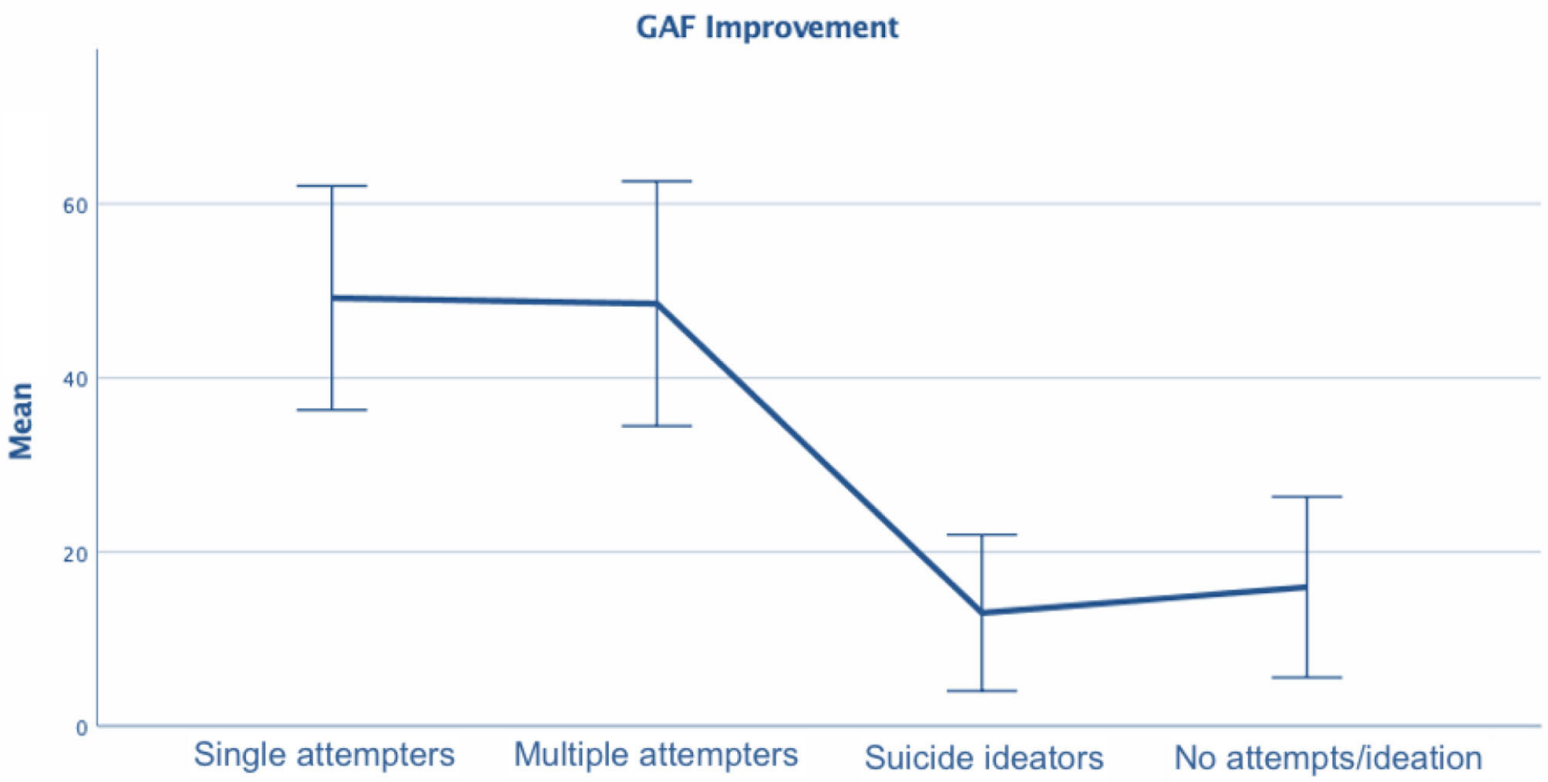
One way ANOVA - global assessment of functioning among subgroups.

**Figure 2:**
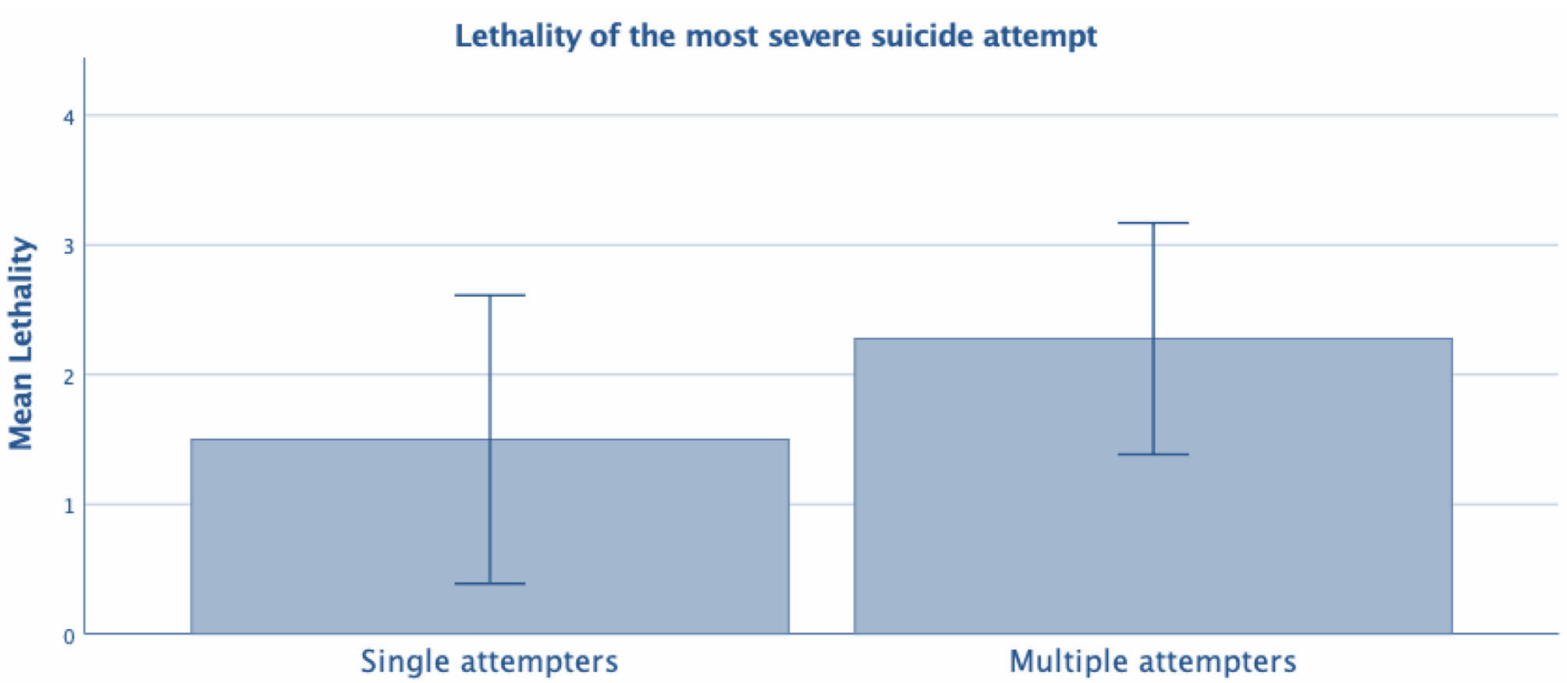
Test *t*- Lethality of the most severe suicide attempt between single vs. multiple attempters.

A multinomial logistic regression model with groups as a criterion that used significant variables at bivariate analysis as independent variables explained 66% of the between-group variance (Nagelkerke *R*^2^ = 0.657; −2LL = 522.69; $\chi_{18}^{2}$ = 324.81, *p* < 0.001) (not reported in the tables). Overall, GAF improvement ($\chi_{3}^{2}$ = 285.64, *p* < 0.001), number of previous hospitalizations ($\chi_{3}^{2}$ = 8.39, *p* < 0.05; not significant in single comparisons), and DUI ($\chi_{3}^{2}$ = 8.45, *p* < 0.05) were significantly and independently associated with group differences. Marital status ($\chi_{6}^{2}$ = 4.64, *p* = 0.59) and diagnosis ($\chi_{3}^{2}$ = 1.91, *p* = 0.59) were not associated with group differences. Compared to controls, SSAs were more likely to have higher GAF improvement (OR=1.20; 95% CI = 1.15/1.25) and a longer DUI (OR=1.09; 95% CI = 1.03/1.16). MSAs were more likely to have higher GAF improvement as compared to controls (OR=1.20; 95% CI = 1.15/1.25). Patients with SI and controls did not differ on any variables (Figures 1, 2)

## Discussion

The present study sought to analyze the most relevant differences in sociodemographic and clinical characteristics between SSAs and MSAs. Comparisons were also made between suicide attempters and two subgroups of controls (patients with SI and psychiatric patients with no suicide risk). Overall, we found a few differences between MSAs and SSAs. MSAs were more frequently single and divorced/widowed, and less frequently married. These differences support existing data on the relationship between unmarried status and high suicide risk (29). In addition, MSAs had a higher lethality of the most severe suicide attempt. In MSAs, the absence of a stable relationship might represent a risk factor for the use of dysfunctional coping mechanisms in the presence of negative life events. For example, a previous study by Pompili et al. (14) found a high presence of at least one stressful life event both throughout childhood/adolescence and within the last 6 months in MSAs vs. SSAs (42.6 vs. 33.8%). Pompili et al. (14) did not find any differences between suicide attempters and non-attempters in terms of marital status but found that more SSAs (63.9%) reported low social support than MSAs (48.9%). However, these contrasting results can be attributed to the different samples analyzed, as well as to differences in research methodologies and designs between studies.

A higher lethality of the most severe suicide attempt in MSAs is also consistent with previous studies that have shown a positive correlation between the number of attempts and the lethality of attempts (30). The present study found that MSAs had higher suicide lethality than SSAs, which is in line with existing research supporting the hypothesis that MSAs constitute a subgroup of suicide attempters, particularly at risk of completing suicide, with a distinctive clinical phenotype and a higher lethality of attempts (31). In contrast, Michaelis et al. (6) reported higher suicide lethality in SSAs as compared to MSAs.

Previous studies also investigated clinical differences between SSAs and MSAs, focusing on patients sharing the same psychiatric diagnosis (6, 15). Boisseau et al. (32) found that Axis I disorders were not predictive of repeat suicidal behavior in a 10-year follow-up study. Notably, SSAs and MSAs differed only in terms of the diagnosis of borderline personality disorder.

In our study, only GAF improvement and DUI were independently associated with group differences after multivariate analysis. Suicide attempters generally reported higher GAF improvements than controls. This result could be associated with the scoring procedure of the GAF scale, which gives a score of 10–1 in the presence of a highly lethal suicide attempt while suggesting higher scores in the presence of severe psychopathology (e.g., hallucinations and delusions). In a naturalistic study, Altamura et al. (33) investigated factors associated with a longer DUI in 320 patients with bipolar disorders. The authors reported a higher frequency and number of suicide attempts in those with a longer DUI when compared to those with a shorter DUI. However, different results were reported by Dell'Osso et al. (34), who investigated sociodemographic and clinical variables characterizing patients with bipolar disorder and a prior suicide attempt. Furthermore, a longer DUI may negatively influence the clinical course and the response to treatment of several psychiatric diagnoses (35, 36) often associated to suicide risk. However, larger prospective studies are warranted to further investigate the role of the DUI within suicide risk. Suicide attempters had a higher rate of personality disorders than controls with no suicide risk. This finding is in line with previously published studies (15, 37) reporting an association between repeat suicidal behaviors and personality disorders, mainly borderline personality disorder. Of note is the fact that the proximal risk factor may play an important role in the precipitation of suicide (38). Traumatic experiences, as in the case of natural disaster and health emergency as in the case of pandemics, may act as a major stress in the vulnerable individual and contribute to a higher risk of attempting suicide (39, 40).

The results of the present study indicated that patients with SI and controls did not differ on any variables. This might be related to the fact that SI seriousness was not assessed using distinct psychometric instruments (41). Unfortunately, SI, especially when manifesting with mild features, is common and it may not always be possible to distinguish ideators from controls. The present results, however, showed that ideators and attempters need to be considered as two distinct populations. More research is needed to understand how and to what extent these sociodemograhic and clinical differences are able to characterize attempters and ideators (42).

## Limitations

The present study needs to be considered in light of the following shortcomings that limit the generalizability of the present results. First, the sample size is relatively small and may not be representative of all SSAs and MSAs. Second, Italian law dictates that acute psychiatric patients admitted to an emergency department be hospitalized in a psychiatric ward, which is generally part of a public hospital. An array of clinical states and circumstances are commonly part of daily clinical practice, but a systematic assessment using psychometric instruments and a homogenous approach of psychiatrists working in the psychiatric unit are lacking. Finally, the subjects in this study were all inpatients and several suicide attempters who made non-lethal attempts might not be hospitalized; this may limit the generalizability of the study. Furthermore, we do not know whether there were differences between subjects who participated and subjects that did not participate in the study.

Finally, the cross-sectional nature of the present study design should be considered an additional caveat.

## Conclusions

In the present study, MSAs showed higher lethality of their last suicide attempt as compared to SSAs. Moreover, MSAs had distinct sociodemographic characteristics as compared to SSAs and patients with SI. The present results suggest that identifying MSAs could help predict suicide risk and design *ad hoc* prevention strategies, including screening to identify at-risk individuals, public education campaigns, telephone helplines, easy access to psychiatric emergence units, treatment interventions, and follow-up care after suicide attempts.

## Data Availability Statement

The original contributions presented in the study are included in the article/supplementary material, further inquiries can be directed to the corresponding author/s.

## Author Contributions

IB and AF wrote the article. MI provided statistical analysis. BI, BM, SS, ER, DE, GA, and MM collected data. GS, MA, and DL revised the manuscript. MP proposed the idea of the article and revised the entire manuscript. All authors contributed to the article and approved the submitted version.

## Conflict of Interest

The authors declare that the research was conducted in the absence of any commercial or financial relationships that could be construed as a potential conflict of interest.
